# Direct Electrochemical Detection of Tyramine in Beer Samples Using a MWCNTs Modified GCE

**DOI:** 10.3390/s25113322

**Published:** 2025-05-25

**Authors:** Cláudio M. R. Almeida, Maria Fátima Barroso, Manuela M. Moreira, Júlia M. C. S. Magalhães, Luisa Durães

**Affiliations:** 1 University of Coimbra, CERES, Department of Chemical Engineering, 3030-790 Coimbra, Portugal; luisa@eq.uc.pt; 2 LAQV-REQUIMTE, Instituto Superior de Engenharia do Porto, Instituto Politécnico do Porto, Rua Dr. António Bernardino de Almeida 431, 4200-072 Porto, Portugal; manuela.moreira@graq.isep.ipp.pt; 3 LAQV-REQUIMTE, Departamento de Engenharia Química, Faculdade de Engenharia, Universidade do Porto, Rua Dr. Roberto Frias, 4200-465 Porto, Portugal; jmagalh@fe.up.pt

**Keywords:** tyramine sensor, electrochemical determination, beer samples

## Abstract

**Highlights:**

Direct determination of tyramine (TYR) in different beer samples, using an electrochemical method with a GCE electrode modified with MWCNTs. By using SWV and controlled pH conditions, TYR was assessed in beers with basic sample pretreatments to provide an easy and rapid method. The developed method was compared with a standard technique to validate its reliability.

**What are the main findings?**

- Portable electrochemical method for TYR detection with simple sample pretreatment.
- Direct TYR determination in beer samples.

**What is the implication of the main finding?**

- Rapid on-site TYR assessment.
- Quality control and corrective measures implementation.

**Abstract:**

In this study, an electrochemical method is presented for the direct determination of tyramine in beer samples. A multi-walled carbon nanotubes (MWCNTs) modified glassy carbon electrode (GCE) was developed for the detection and quantification of tyramine at a low potential of 0.53 V. The electrochemical process and sensor parameters were thoroughly investigated to establish optimal analysis conditions. The method demonstrated a linear response range from 3 to 9 µM, with a limit of detection (LOD) of 0.34 µM and a limit of quantification (LOQ) of 1 µM. The developed sensor was successfully applied to commercial beer samples for tyramine analysis. The results were compared with those obtained using the standard high-performance liquid chromatography (HPLC) technique, highlighting the sensor’s potential for tyramine determination in aqueous food samples without the need for complex sample preparation.

## 1. Introduction

Food intoxication caused by tyramine (TYR), the so-called “cheese reaction”, is commonly associated with high consumption of cheeses due to their capacity to potentiate sympathetic cardiovascular activity by releasing noradrenaline [1]. A healthy person can consume a tolerable amount up to 200–800 mg per single oral intake [2]. TYR may be involved in the constriction of blood vessels, inhibiting blood flow and increasing blood pressure [3]. It can also increase the tear, salivation and the physiological respiratory rate [4]. Among different food products, high levels of TYR can be found in beers. TYR results from the decarboxylation reaction from the amino-acid precursor tyrosine. However, regardless of the initial levels of tyrosine in beers, this factor did not seem to have significant influence on tyramine formation; on the contrary, the degree of lactic acid bacteria contamination shows a major influence on TYR production [5,6]. Therefore, the search for simple and reliable methods capable of determining these contaminants in foods has been increased [7,8]. Among the different methodologies, electrochemical sensors represent a real alternative [9,10]. Biogenic amines which undergo oxidation/reduction that produces changes in their electrochemical potential are suitable to be analyzed by electrochemical methods.

Different strategies have been described in the literature to develop useful sensors for real applications. One of most used strategies consists of building carbon nanostructures on the surface of the electrodes. This usually leads to large surface area, high adsorption ability and more active sites for the target molecules. Raoof et al. developed a functionalized multi-walled carbon nanotubes (fMWCNTs) modified glassy carbon electrode (GCE) for simultaneous determination of TYR and acetaminophen (Ac) [11]. By using differential pulse voltammetry (DPV), the developed sensor could completely resolve the voltammetric response of Ac and TYR, reaching limits of detection (LOD) of 0.42 µM for Ac and 0.8 µM for TYR. Due to the large surface area, high sorption capacity and numerous active sites of the nanostructures caused an increase in oxidation signals and a shift to lower oxidation potentials. The applicability of the sensor for real samples analysis was assessed for the determination of TYR, by a standard addition method, in yogurt and urine samples. The same authors also reported a MWCNTs modified GCE voltammetric sensor for simultaneous determination of levodopa, uric acid and TYR [12]. The modified electrode properties toward the electrocatalytic oxidation of TYR were studied using voltammetry techniques, offering substantially lower overpotential for TYR electro-oxidation in phosphate-buffer solution (pH 7.0). The LOD for TYR was defined to be 0.647 µM. Furthermore, this sensor showed an ability to be used in routine analysis of TYR in real samples, e.g., urine.

An electrochemical sensor consisting of reduced graphene oxide nanosheets (ERGO) on the surface of a GCE was developed for sensitive detection of octopamine and TYR [13]. The electrocatalytic oxidation of these analytes was investigated using DPV. The obtained sensor was applied for the quantification of octopamine and TYR in concentration ranges from 0.5 to 40 µM and 0.1 to 25 µM, respectively, with good reproducibility, selectivity and stability. In addition, the obtained LODs for octopamine and TYR were 0.1 µM and 0.03 µM, respectively. The developed sensor was successfully applied in the detection of both molecules in commercially available beers.

Meng and Lin have developed a sensor using overoxidized polypyrrole-gold composite film for the detection of TYR in fermented rice wine [14]. The sensor exhibited high sensitivity with an LOD as low as 0.01 μM. The sensor kept the reproducibility over two weeks, which is important for daily operations.

In another approach, a MWCNTs/graphene film over a modified ionic liquid carbon paste electrode was used for electrochemical determination of TYR by the square wave voltammetry technique [15]. The prepared sensor showed an excellent electrocatalytic effect for TYR oxidation. The obtained results can be assigned to the synergistic effect of carbon nanotubes, graphene and ionic liquid. The obtained LOD was set at 0.5 μM.

Other frequently used strategies for electrode modification involve the deposition of macrocycles, monomers or polymer films in the electrode surface. In this way, a TYR sensor was developed using a pillar arene [7] as a surface modification material on a GCE [16]. Pillar arenes are macrocycles composed of hydroquinone or dialkoxybenzene units linked in the para position by methylene bridges [17]. They are structurally like the cucurbiturils and calixarenes, and due to this, they can play an important role in host–guest chemistry. Under optimum experimental conditions (pH = 9 and a working potential of +0.6 V), linear working ranges were set from 0.08 to 1.5 µM with an LOD of 0.04 µM. Analytical applicability of the sensor was tested by TYR analysis and recovery studies in the sauerkraut sample with apparently good performance.

In another work [18], a simple electrochemical method was developed for fast detection of TYR in rice vinegar, resorting to a film of poly(o-aminophenol), an organic amphoteric compound used as a reducing agent, electropolymerized on the surface of the GCE. After electrochemical treatment of the poly(o-aminophenol) film in alkaline solution, the modified sensor showed good electrochemical response to TYR. The oxidation current vs. TYR concentration showed good linear relationship in the range of 0.1 to 200 μM. The LOD was 0.054 μM. The recovery rates of TYR in rice vinegar sample were from 95.6% to 117.2%. The sensor was simple, inexpensive, and suitable for fast detection of TYR in rice vinegar samples.

Following the same approach, electropolymerized toluidine blue was used as a sensor modifier material on carbon screen printing electrodes for the determination of TYR [19]. The toluidine blue-modified SPE showed a TYR oxidation peak at lower potentials (0.67 V). This represents a 25% decrease in the oxidation potential compared to the unmodified electrode (0.9 V). TYR was detected over a wide linear range from 0.02 to 270.5 μM, with the developed sensor presenting a low LOD of 7 nM. Regarding the selectivity to TYR over other biogenic amines, such as dopamine, L-cystine, putrescine, and histamine, which are commonly found in real samples, the sensor does not show a significant change in current for the studied interfering molecules, even at high concentrations.

Despite the available information about the electrochemical oxidation of TYR, the definition of a methodology to assess this contaminant in a real scenario is still not well established. In this work, a TYR sensor based on non-functionalized MWCNTs modified GCE and the analysis conditions were investigated to develop a methodology for TYR determination in beer samples.

## 2. Materials and Methods

### 2.1. Chemicals, Reagents and Standard Solutions

Buffer solutions were prepared from phosphate sodium salts in the pH range from 4 to 9. Di-potassium hydrogen orthophosphate anhydrous (>99%) and potassium dihydrogen orthophosphate (>99.5%) were obtained from Fisher Chemicals (Pittsburgh, PA, USA) and used as received. TYR (≥98.0%), 1,7-diaminoheptane (98%), used as internal standard (IS), and dansyl chloride (Dns-Cl, ≥99%), used as derivatization reagent, were purchased from Sigma-Aldrich (Saint Louis, MO, USA). Acetonitrile (99.9%) was purchased from Carlo Erba (Cornaredo, MI, Itália), acetone (≥99.8%) was acquired from Emsure from Merk, (Darmstadt, Germany), and hydrochloric acid (HCl, 37% *w*/*v*) and perchloric acid (PCA, 70% *w*/*w*) were purchased from Sigma-Aldrich. All the chromatographic solvents were filtered through a 0.22 µm nylon membrane (Fioroni Filters, Ingré, France) using a vacuum pump (Dinko D-95, Barcelona, Spain) and degassed for 15 min in an ultrasonic bath (Sonorex Digital 10P, Bandelin DK 255P, Berlin, Germany). Before the chromatographic analysis, sample extracts were filtered through 0.22 µm PTFE syringe filters (Sigma-Aldrich, Saint Louis, MO, USA). The MWCNTs (0.05 mg mL^−^^1^ in dimethylformamide (DMF)) were obtained from Metrohm (Herisau, Switzerland). Ultra-pure water was used to prepare all the aqueous solutions. Stock solutions of 1,7-diaminoheptane (IS) and TYR were prepared in 0.1 mol L^−^^1^ HCl at a concentration of 1000 mg L^−^^1^. Working solutions were prepared in acetonitrile from the stock solutions, and stored at 4 °C protected from light. The Dns-Cl solution (0.5% *w*/*v*) was prepared daily in acetone.

### 2.2. Electrode Preparation

**Scheme 1:**
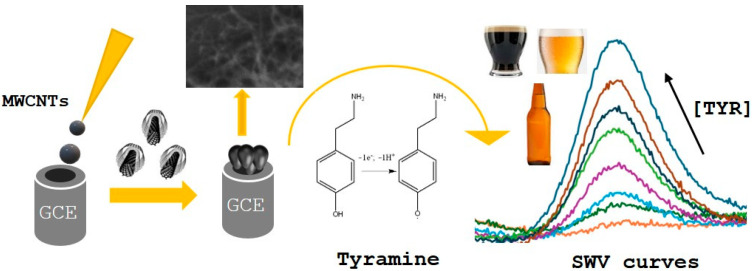
Illustration of the sensor preparation and working principle.

Prior to modification, the GCE was polished with 0.3 and 0.05 µm alumina slurries using a polishing cloth. Then, the electrode was rinsed thoroughly with ultra-pure water and sonicated in ethanol and water for 5 min. After that, 15 µL of MWCNTs suspension in DMF was dropped in the electrode working surface and left 1 h at 27 °C for solvent evaporation. Before use, the electrode was rinsed with ultra-pure water. The schematic diagram of the preparation and analysis methods is shown in Scheme 1.

### 2.3. Surface Characterization

The morphology and microstructure of the MWCNTs on the GCE surface was observed by a high-resolution Compact/VP Compact field-emission scanning electron microscope (FESEM) (Zeiss Merlin, Oberkochen, Germany). After drop-casting the MWCNTs solution on the top of the GCE and leaving it to dry for 24 h, the electrode was placed in the sample holder and observed without any metal sputtering.

The study of the surface modification efficiency was performed also by voltammetry and electrochemical impedance spectroscopy (EIS) using a potentiostat/galvanostat Gamry 100 T equipment (Gamry Instruments, Warminster, PA, USA). The EIS measurements were recorded between 0.1 and 65,000 Hz in the potentiostatic mode.

### 2.4. Electrochemical Analysis

All electrochemical studies were performed in a potentiostat/galvanostat Gamry 100 T equipment (Gamry Instruments, USA) and the obtained data were analyzed using EchemAnalyst 2, version 7 software. For the electrochemical cell, the prepared MWCNTs-GCE was used as a modified working electrode, and a saturated Ag/AgCl electrode and platinum wire were used as a reference and counter electrode, respectively. Regarding the square wave voltammetry (SWV) experiments, the voltammograms were collected between 0.2 and 1 V, using a pulse size of 25 mV and a frequency of 50 Hz. An equilibration time of 5 s was applied.

### 2.5. Beer Samples Preparation

Commercial beer samples (from Super Bock, Matosinhos, Portugal) were purchased from local supermarkets. The beer was filtered using a 45 µL filter syringe to degas and any solid present in the sample was removed. Then, 1 mL of each sample was mixed with 19 mL of phosphate buffer at pH 9.2. The determination method adopted was direct determination, by comparing the obtained peak current with the calibration curve. Two types of beer were used: black and blond. These samples were selected because they are different types of beer for one of the most consumed brands in Portugal and they exhibit very different amounts of tyramine.

### 2.6. Tyramine Analysis by HPLC-FLD

Prior to derivatization reaction, beer samples were degassed in an ultrasonic bath for 30 min. The derivatization of beer samples or TYR standard followed the procedure previously described by Angulo et al. (2020) [20], with some modifications. Briefly, 5 mL of beer or TYR standard were mixed with 200 µL of NaOH 10 M and 1 mL of 0.5 M carbonate-hydrogen carbonate buffer (pH 10.5). Then, 1 mL of this mixture was collected and mixed with 2 mL of Dns-Cl solution 0.5%. The mixture was left in a termobloc (J.P. SELECTA) at 40 °C for 15 min and then cooled down in the dark at room temperature for 30 min. Afterwards, 50 µL of ammonium hydroxide (25%) was added to remove the surplus of Dns-Cl. Then, the mixture was made up to 5 mL with acetonitrile and centrifuged (5 min, 3000 rpm, 4 °C). Prior to HPLC analysis, the supernatant was filtered through a 0.22 µm PTFE filter.

The HPLC-FLD analysis was performed on a LC Shimadzu LC-20AD Prominence equipped with a SIL-20 AHT autosampler, a CTO-10 AS VP oven and a RF-20 AXS fluorescence detector all from Shimadzu Corporation (Tokyo, Japan). The HPLC method was based on the one previously described by Herrero et al. (2016) [21], with some modifications. A Luna C18 column (150 mm × 4.6 mm, 5 µm) from Phenomenex was used for the separation of the derivatized TYR and beer samples. The injection volume was 10 µL and the mobile phase was composed of water (A) and acetonitrile (B). The gradient program applied was as follows: 0–6 min, 60–70% (B); 6–8 min, 70% (B); 8–13 min, 70–95% (B); 13–20 min, 95% (B); 20–25 min, 95–60% (B); 25–30 min, 60% (B). The column temperature was 30 °C and the flow rate was 1 mL/min. The excitation and emission wavelengths were 350 nm and 520 nm, respectively.

## 3. Results

### 3.1. Surface Characterization of MWCNTs/GCE by CV, SEM and EIS

**Figure 1:**
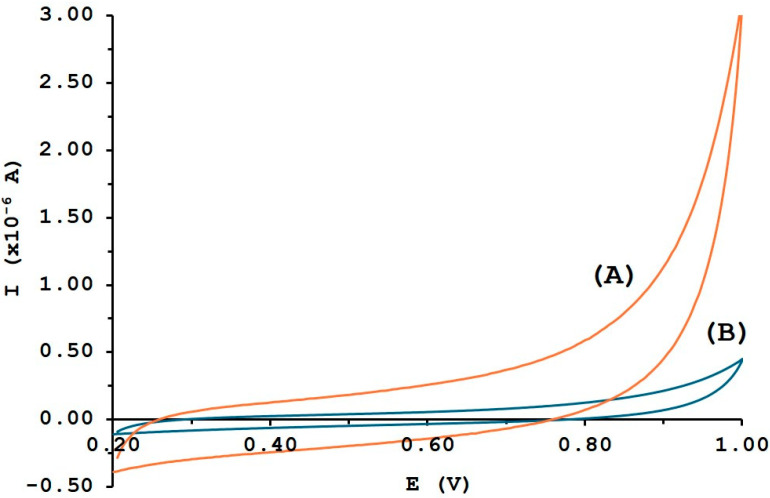
Cyclic voltammograms of the blank signal obtained in phosphate buffer with (A) MWCNTs-GCE and (B) naked GCE.

Measurements of the background noise generated when using a naked GCE and a MWCNTs/GCE were performed using CV in a phosphate buffer at pH 9.2 (Figure 1). Higher conductivity and lower resistivity were observed when the GCE was modified with MWCNTs.

**Figure 2:**
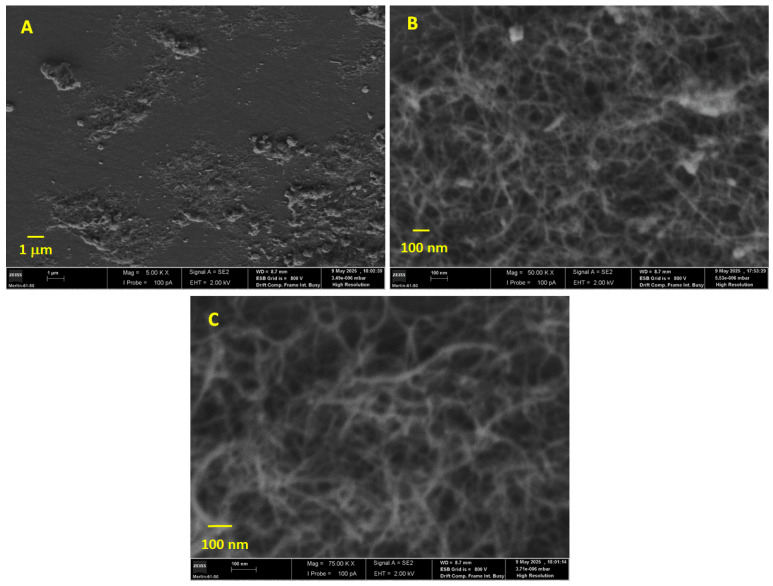
SEM images of the MWCNTs on the top surface of the GCE with a magnification of (**A**) 5000×, (**B**) 50,000× and (**C**) 75,000×.

High-resolution images of the MWCNTs/GCE were captured using a FE-SEM to correlate the voltammetry response to the surface morphology. These images are displayed in Figure 2. As observed in Figure 2A–C, a compact layer of MWCNTs covering the GCE was obtained. As the MWCNTs are elastic, they can bend, roll and twist, forming a porous and three-dimensional structure that produces a high-conductive sensor. Also, the presence of the MWCNTs increase the active surface area for target detection. The observed MWCNTs are quite long and show a diameter of approximately 14 to 19 nm.

**Figure 3:**
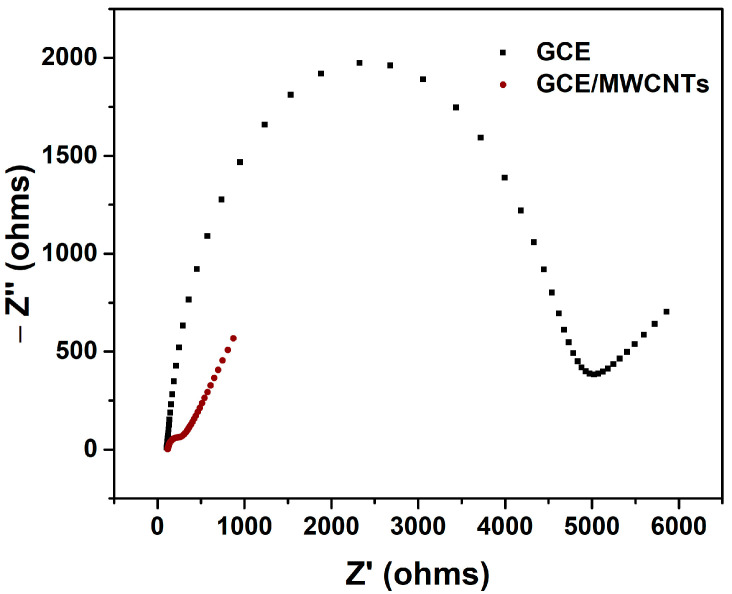
Nyquist plots of bare GCE and MWCNTs/GCE in 5.0 mM of [Fe(CN)_6_]^3−/4−^ containing 0.1 M KCl solution.

Electrochemical impedance spectroscopy (EIS) was performed to study the surface modification mechanism, which is based on the impedance changes promoted by the electron transfer resistance (Rct). The Rct values of the GCE and MWCNTs/GCE were calculated using a 5 mM of a [Fe(CN)_6_]^3−/4−^ solution containing 0.1 M KCl. Figure 3 presents the Nyquist diagram where the diameter of the semicircle is proportional to the Rct, and the linear portion is correlated to the diffusion process.

From the diagram, it is clear that the MWCNTs/GCE exhibited a smaller semicircle in comparison with the bare GCE, indicating that the modified GCE has high conductivity. A calculated Rct value of 4.67 kΩ for the GCE emphasizes the low electron transfer rate, whereas the MWCNTs/GCE shows a Rct value of 178.3 Ω, indicating the synergistic effect of conductivity due to the MWCNT adsorbed layer and consequent rapid electron transfer in the redox probe.

### 3.2. Evaluation of the Electrode Surface Area

**Figure 4:**
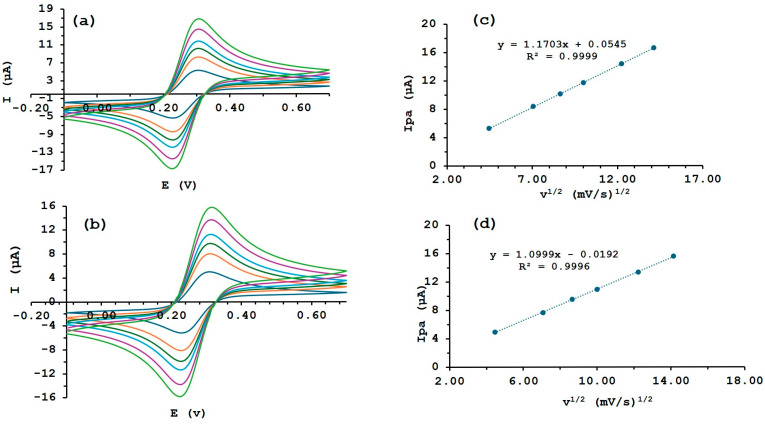
Cyclic voltammograms of a 1 mM [Fe(CN)_6_]^3−/4−^ solution with 0.1 M KCl, at scan rates from 0.02 to 0.2 V s^−1^, in (**a**) MWCNTs/GCE, (**b**) GCE. Plot of *I*_pa_ vs. *v*^1/2^ for the (**c**) MWCNTs/GCE and (**d**) GCE.

The GCE surface characterization was performed by using electrochemical redox probe solutions of [Fe(CN)_6_]^3−/4−^ in KCl. For that, CV was performed on the naked GCE and on the MWCNT/GCE (Figure 4). Immobilizing MWCNT onto the GCE resulted in a stable enhancement in current responsiveness, demonstrating that the electrochemical active sites of GCE increased when the GCE surface was modified with MWCNT. The MWCNT/GCE exhibits higher peak current response (*I*_pa_ = 11.7 µA) compared to the naked GCE (*I*_pa_ = 10.9 µA) at 100 mVs. These differences are attributed to electrocatalytic activity and surface area enhancement by surface modification through the MWCNT. The electrochemical active surface area of the GCE and MWCNTs/GCE was calculated by analyzing the cyclic voltammograms (Figure 4a,b) obtained for the electrochemical redox [Fe(CN)_6_]^3−/4−^, at scan rates ranging from 20 to 200 mVs^−1^. The plot of *I*_pa_ versus *v*^1/2^ shows linearity with *R*^2^ of 0.9992 for the MWCNTs/GCE and 0.9996 for the GCE. The active electrochemical surface area was estimated considering the slope of *I*_pa_ versus *v*^1/2^ (Figure 4c) based on the Randles–Sevcik equation [22,23,24]:

(1) $$I_{\mathrm{pa}}=2.69\times 10^{5}\;n^{3/2}\;A\;D^{1/2}\;C\;\nu^{1/2}$$

where *I*_pa_ represents the anodic peak current (Amperes), *n* is the number of electrons exchanged during the redox process (presumed to be equal to one), *A* represents the surface area of the electrode (cm^2^), *D* is the diffusion coefficient of the 1 mM [Fe(CN)_6_]^3−/4−^ in 0.1 M of KCl, considered to be equal to 7.6 × 10^−6^ cm^2^ s^−1^, *C* represents the concentration of the electrochemical redox probe (mol cm^−3^), and *v* represents the scan rate (V s^−1^). The electroactive surface area was maximum for the MWCNTs (0.050 cm^2^) in comparison with the naked GCE (0.047 cm^2^).

### 3.3. Electrochemical Behavior of Tyramine

**Figure 5:**
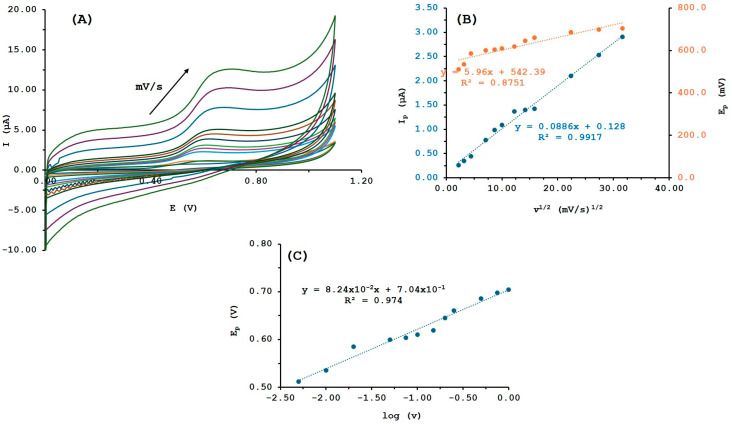
(**A**) Cyclic voltammograms of 50 µM TYR at the MWCNTs in phosphate buffer at pH 9.2. (**B**) Plot of *I*_pa_ vs. *v*^1/2^. (**C**) Plot of *E*_pa_ vs. log (*v*).

The CV technique was used to explore the oxidative behavior of 50 µM TYR in the MWCNTs/GCE using PBS buffer at pH 9.2 (Figure 5). The cyclic voltammograms of TYR presented a single irreversible anodic peak at + 0.527 V vs. AgCl/Ag and no reduction peak was observed in the cathodic direction, indicating the irreversible nature of the electrode reaction. Although the modification with MWCNTs is not particularly relevant in the peak current, the peak potential is significantly reduced in ca. 60 mV. This is relevant to detect TYR at lower potentials.

The influence of the scan rate on the anodic peak current was studied within the range 0.005–1.00 V s^−1^ (Figure 5A). The cycles were carried out within the increased values of scan rates and produced a linear relationship with the *v*^1/2^, indicating that the electrochemical process at the surface of the electrode was mainly controlled by diffusion [23,24] (Figure 5B): (*I*_pa_ (µA) = 8.86 × 10^−2^ *v*^1/2^ (mVs^−1^)^1/2^ + 0.128; *R*^2^ = 0.9917).

In addition, the reaction is irreversible due to the linear relationship between the anodic peak potential (*E*_pa_) and the logarithm of scan rate (*E*_pa_ = 8.24 × 10^−2^ log(*v*) + 7.04 × 10^−1^ with *R*^2^ = 0.974, *n* = 12; data from three independent assays) (Figure 5C). For an irreversible process, the Laviron equation (Equation (2)) can be used to determine the number of electrons (z) involved in the oxidation reaction of TYR [22].

(2) $$E_{\mathrm{pa}\;}=E_{0\;}+\left(\frac{2.3RT}{\alpha zF}\right)\log\left(\frac{RTk^{0}}{\alpha zF}\right)+\left(\frac{2.3RT}{\alpha zF}\right)\log v$$

where α is the electron transfer coefficient, *k*^0^ is the standard rate constant of the reaction, *R* is the gas constant (8.314 J K^−1^ mol^−1^), *F* is the faraday constant (96,480 C mol^−1^), and *T* is the absolute temperature (298 K). The slope of the *E*_pa_ versus log(*v*) (equal to 2.3*RT*/*αzF*) was used to obtain αz which corresponds to 0.717. Considering α is equal to 0.5, the number of electrons involved in the reaction is 1.43.

### 3.4. Effect of pH

**Figure 6:**
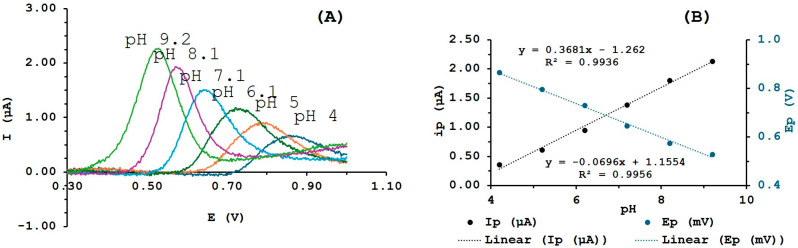
(**A**) SWV obtained for 50 µM of MWCNTs in phosphate buffer, in the pH range 4.2–9.5; (**B**) plot of the *E*_pa_ vs. pH and plot of *I*_pa_ versus pH of the TYR solution.

After observing the electrochemical oxidation of TYR at the MWCNTs/GCE, a more sensitive and rapid voltammetric technique, the square wave voltammetry (SWV), was used to assess the effect of the pH on the TYR peak shape and height. The effect of changing the pH of the medium on the redox response of TYR at MWCNTs/GCE was examined in PBS buffer over the range from 4.2 to 9.2. As illustrated in Figure 6, *E*_pa_ shifts linearly to lower values as pH increases from acidic to basic medium.

The plot of *E*_pa_ versus pH (Figure 6B) evidently specifies that *E*_pa_ linearly depends on pH values presenting an equation *E*_pa_ (V) = −0.0696 pH + 1.1554, and a correlation coefficient of *R*^2^ = 0.9956. The slope calculated of 69.9 mV/pH unit is nearly the theoretical Nernstian slope values. The number of protons (*P*) involved in the electrochemical oxidation of TYR can be calculated by using a mathematical equation modulated specifically for irreversible diffusion-controlled system applying Equations (2)–(4) [19,23,24]:

(3) $$E_{\mathrm{pa}}-E_{\mathrm{pa}/2\;}=\frac{0.048}{\propto n}$$

where *E*_pa_ and *E*_pa/2_ are the anodic peak potential and the half-wave anodic peak potential, and *αn* the charge transfer coefficient. Using Equations (3) and (4) the value of *αn* and the number of protons were calculated, being *αn* = 0.954 and the number of protons = 1.13. Moreover, using Equation (5), a value of the diffusion coefficient for a 50 µmol L^−1^ TYR solution of 1.4718 × 10^−5^ cm^2^ s^−1^ was calculated.

(4) $$\frac{\Delta E_{\mathrm{pa}}}{\Delta pH}=-\frac{59\;P\;}{\alpha n}$$

(5) $$I_{\mathrm{pa}}\;=\;2.99\times 10^{5}\;n\;{\left(\alpha n\right)}^{1/2}\;A\;D^{1/2\;}C\;{\nu \;}^{1/2}$$

The observed *I*_pa_ increased as the pH increase from 4.2 to 9.2 (Figure 6B). It is clear that the higher peak current was obtained at pH 9.2. Furthermore, at this pH a narrow peak is also obtained, and the oxidation potential of TYR is observed at lower potentials.

**Scheme 2:**
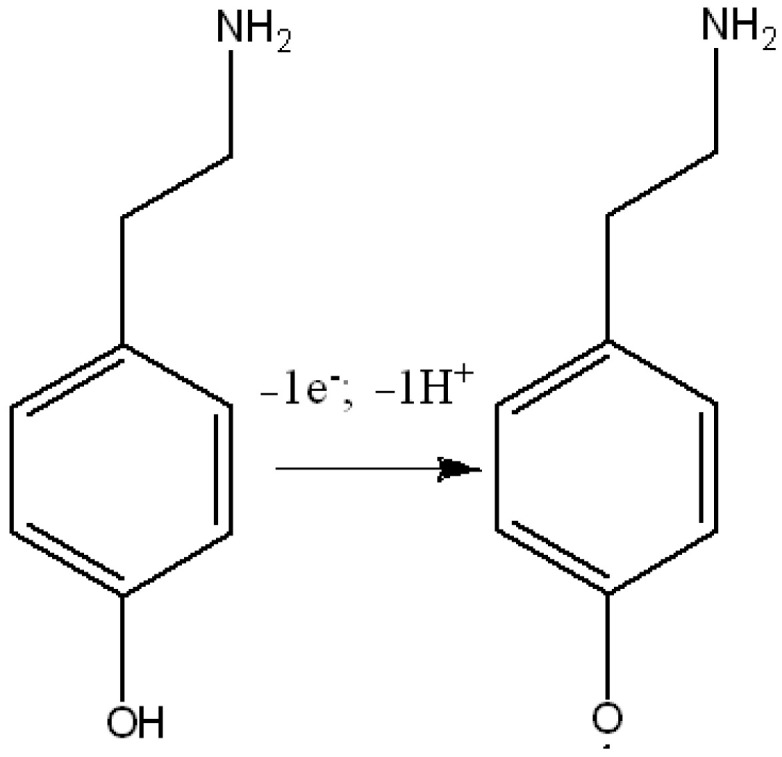
Probable electrochemical mechanism of TYR oxidation.

The probable electrochemical oxidation mechanism is depicted in Scheme 2. The oxidation peak is due to the oxidation of TYR, which involved a proton and electrons, in this case 1.43 electron and 1 proton.

In the case of aromatic amines, electrochemical oxidation mostly involves radical cations, and the covalent attachment to the electrode surface was not observed [25]. Aliphatic amines are more difficult to oxidize and originate unstable intermediates due to the lack of delocalization of the charge. For this reason, they are not investigated since the oxidation potentials are much higher. The attachment of primary amines to carbon surfaces is easier compared to secondary or tertiary amines. These variations are attributed to the differences in steric hindrance at the radical cation level. However, with the exception of alcohol oxidation, all other known reactions are driven by the reactivity of radicals [26].

The increase on pH, usually results in a decrease in the oxidation potential of the amine groups [27]. Under the present conditions (pH = 9.2), the alcohol and amine groups will be oxidized at lower potentials, which can justify the number of electrons/protons involved in the oxidation mechanism of TYR.

**Figure 7:**
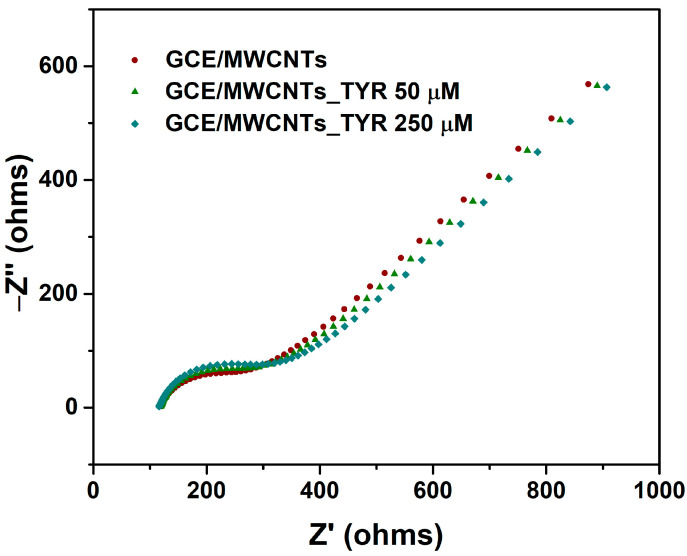
Nyquist plots of the MWCNTs/GCE in 5.0 mM [Fe(CN)_6_]^3−/4−^ solution containing 0.1 M KCl without and with TYR.

As shown before (Figure 3), compared to the bare GCE, the modified MWCNTs/GCE shows a significant decrease in the charge transfer resistance, confirming the attachment of the nanotubes in the sensor surface. Also, the charge transfer resistance increases as the concentration of TYR increases (Figure 7), caused by the increased binding of the TYR molecules on the sensor surface, which decreases the electron transfer [28].

The increase in the charge transfer resistance values from 178.3 Ω to 195.7 Ω and then to 215.8 Ω after TYR addition may also be correlated with the blocking of free spaces on the electrode surface.

### 3.5. Interference Studies

**Figure 8:**
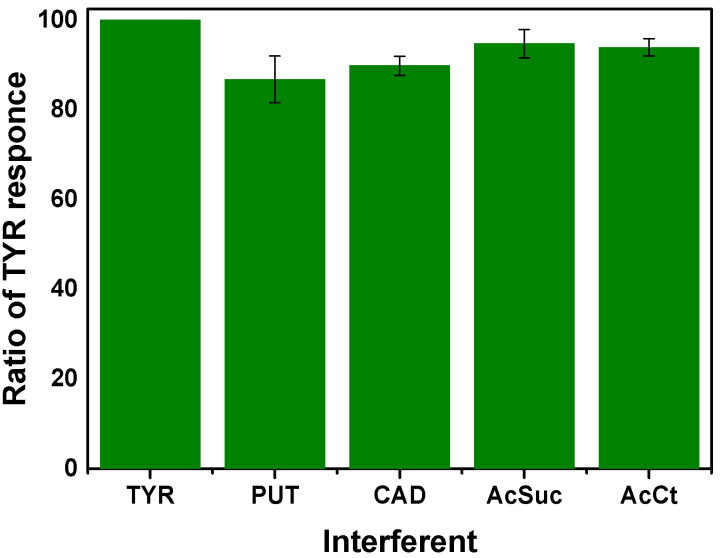
Response for TYR solutions (50 µM) in the presence of different interfering species.

In beer samples, there may be many substances that can interfere with tyramine analysis [29]. After the sensor optimization, various substances that can potentially interfere with tyramine response were investigated, such as putrescine (PUT), cadaverine (CAD), citric acid (AcCit) and succinic acid (AcSu). All solutions were prepared in 0.1 M PBS (pH 9.2). Figure 8 shows the results of the interference study performed toward the electrochemical TYR response.

In general, the results indicate no significant differences in the current. These interfering compounds do not show a significant change in the current response to tyramine since they are oxidized at higher potentials compared to tyramine. However, the presence of PUT seems to have more influence in the TYR detection, with the obtained current ratio falling 13% in the presence of this interfering compound. Based on these results, the MWCNTs/GCE showed excellent selectivity toward TYR.

### 3.6. Stability Studies

**Figure 9:**
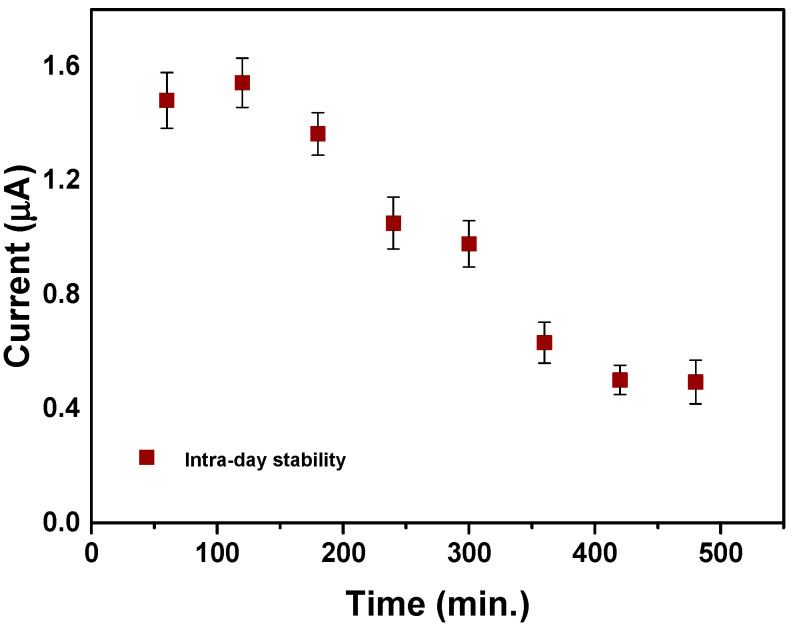
Short-term stability test performed over a period of 8 h. TYR concentration of 50 µM.

A short-term study regarding TYR response was also carried out. The performance of the developed sensor was tested every hour for 8 h within a day (Figure 9).

From Figure 9, it can be concluded that the electrochemical TYR response decreased significantly after three measurements; after this, the TYR response presents a 30% decrease, reaching 67% decrease after seven measurements. This can be due to the TYR adsorption on the sensor surface, limiting subsequent uptake. However, due to the easy preparation of the MWCNTs/GCE sensor, this is not a crucial factor.

### 3.7. Analytical Applications

**Figure 10:**
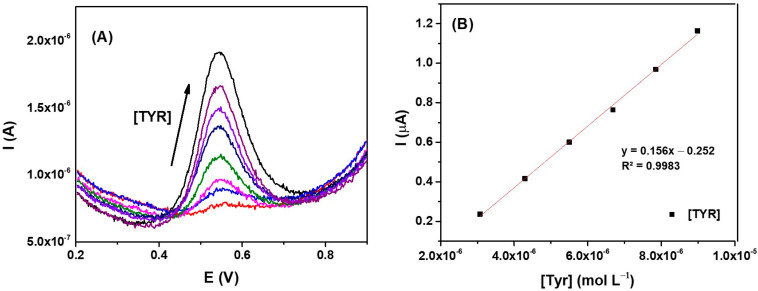
(**A**) SWV curve obtained for TYR concentrations of 0.59, 1.59, 3.07, 4.29, 5.50, 6.69, 7.85 and 8.98 µM. (**B**) Calibration curve for the TYR at pH 9.2 in phosphate buffer.

Quantitative analysis of TYR was accomplished by using square wave voltammetry (SWV) in the range of TYR concentrations from 1 to 9 µM. From Figure 10, it is obvious that the peak intensity shows enhancement with increasing concentration.

Using the optimized conditions, the calibration curve (Figure 10A) was plotted for TYR concentration from 3 to 9 µM, presenting a linear equation: *I*pa (µA) = 0.156 [TYR] (µM) − 0.252, *R*^2^ = 0.9983. The LOD and limit of quantification (LOQ) were 0.34 and 1 µM, respectively, which were comparable to the values reported by other authors (Table 1) [30].

The developed MWCNTs/GCE sensor was applied to determine TYR in beverage samples. Furthermore, it has been described that TYR is present in many foods including fermented alcoholic beverages. One of the products presenting high TYR concentrations is beer [31]. In this way, as proof of concept, commercial beer samples, black and blond, were tested for TYR and the obtained values were compared with those determined by a reference HPLC-FLD method. Table 2 presents the obtained values.

The ability of the MWCNTs/GCE to detect and determine the TYR concentration in both beer samples is evident. The black and blond beers were found to have TYR concentrations of 1.58 × 10^−4^ and 9.02 × 10^−5^ M, respectively, with the black beer having a higher TYR content than the blond. The beer samples were also subjected to HPLC-FLD analysis in order to validate the developed sensor. As is shown in Table 2, there was a strong correlation between the TYR levels and those determined by HPLC for the dark beer samples. However, the results of testing the blond beer showed less accuracy. So, it is possible to conclude that this sensor is sensible to quantify beverages samples presenting high levels of TYR, but have less sensitivity to determine trace amounts of TYR..

## 4. Conclusions

In this work, an easy, versatile and low-cost electrochemical MWCNTs/GCE sensor was constructed for the sensitive and selective TYR detection at pH 9.2.

The electrochemical TYR behavior at the MWCNTs/GCE sensor was studied by CV and SWV techniques. It was found that TYR presents an irreversible oxidation peak at +0.53 V and its oxidation is mainly controlled by diffusion. Furthermore, the TYR electrochemical oxidation is affected by pH, being the anodic peak potential shifted to lower values as pH increases from acidic to basic medium.

Surface morphological studies were performed by CV, FESEM and EIS techniques, which indicated that the presence of MWCNTs on the GCE surface increased the electroactive surface area and the conductivity, promoting a rapid electron transfer.

This electrochemical MWCNTs/GCE sensor was successfully used to detect and quantify tyramine in beer samples. In fact, this sensor showed a great potential for tyramine determination in beverage samples, avoiding complex samples preparation, which is a major advantage for on-site measurements in quality control operations.

The MWCNTs/GCE presented high sensitivity, good reproducibility and a LOD of 0.34 µM. Additionally, the method’s high selectivity along with rapid responsiveness offer additional distinguishing characteristics.

## Figures and Tables

**Table 1:** Comparison of different electrochemical sensors for determination of TYR.

System	Detection Technique	LOD (µM)/LOQ (µM)	Linear Range (μM)	Ref.
fMWCNT/GCE	DPV	0.8/n.a.	Up to 85	[11]
fMWCNTs/GCE	DPV	0.647/n.a.	0.70–75.0	[12]
rGO/GCE	DPV	0.03/n.a.	0.1–25	[13]
Au/PPyox/GCE	ChrAmp	0.027/n.a.	0.2–1200	[14]
fMWCNTs/graphene film/CPE	SWV	0.5/n.a.	1–1000	[15]
Overoxidized Poly(o-aminophenol)/GCE	SWV	0.054/n.a.	0.1–10	[18]
Poly(Toluidine Blue)/CarbonSPE	DPV	0.007/n.a.	0.02–270.5	[19]
MWCNTs/GCE	SWV	0.34/1	3–9	This Work

**Table 2:** TYR determination on commercial beer samples using HPLC-FLD and the developed MWCNTs/GCE sensor.

Beer Sample	HPLC (M)	RD (%)	MWCNTs/GCE (M)	RSD (%)
black	1.65 × 10^−4^ ± 0.04 × 10^−4^	0.05	1.58 × 10^−4^ ± 0.34 × 10^−4^	18.3
blond	5.27 × 10^−5^ ± 0.28 × 10^−5^	0.02	9.02 × 10^−5^ ± 0.88 × 10^−5^	9.7

Beer Sample

HPLC (M)

RD (%)

MWCNTs/GCE (M)

RSD (%)

## Data Availability

Data are available on request.
